# Material Flow Control and Process Design in Constraint Ring Rolling of Thin-Walled Conical Cylinders with Three Ring Ribs

**DOI:** 10.3390/ma18061262

**Published:** 2025-03-13

**Authors:** Duanyang Tian, Xinghui Han, Zhuwei Lu, Wuhao Zhuang, Zhaosen Zhang, Zushen Deng, Lin Hua

**Affiliations:** 1 Hubei Key Laboratory of Advanced Technology for Automotive Components, Wuhan University of Technology, Wuhan 430070, China; tianduanyang@whut.edu.cn (D.T.); 341774@whut.edu.cn (Z.L.); zhangzhaosen2025@163.com (Z.Z.); 18376674977@163.com (Z.D.); hualin@whut.edu.cn (L.H.); 2 Hubei Longzhong Laboratory, Xiangyang 441000, China

**Keywords:** conical cylinders with three ring ribs, constraint ring rolling, unreasonable material flow, process optimization

## Abstract

Thin-walled conical cylinders with three ring ribs (TWCCTRR) are the critical bearing-load components of aerospace equipment, and now the high-performance fabrication of TWCCTRR is confronting great challenges. Constraint ring rolling (CRR) is a new plastic-forming technique that shows great potential in forming high-performance TWCCTRR. However, unreasonable material flow (UMF) is prone to occur in the CRR of TWCCTRR, which weakens its performance. Therefore, the problem of UMF in the CRR of TWCCTRR is investigated in this work. Through finite element (FE) simulation, it is found that UMF occurs at the bottom of the middle rib in single-pass CRR of TWCCTRR because the rib at the middle part is the earliest among the three ribs to be completely filled. Therefore, the double-pass CRR process is proposed for forming TWCCTRR without UMF, which makes the three ribs fully fill simultaneously to avoid UMF. Based on the FE simulation, in contrast to the single-pass CRR, the deformation homogeneity of TWCCTRR obviously improved, and meanwhile, the maximum radial forming force is approximately reduced by 15% in double-pass CRR due to the variation in material flow mode. Investigation results offer theoretical guidance for the CRR of high-performance TWCCTRR.

## 1. Introduction

Thin-walled conical cylinders with three ring ribs (TWCCTRR) are critical light-weight carrying components of aerospace equipment, and they must obtain high performance so as to carry heavy loads under extreme external environments. TWCCTRR, generally containing one thin conical skin and three high ring ribs, show extreme geometry, which makes it hard to manufacture high-performance TWCCTRR. Hence, TWCCTRR is now mainly manufactured by the cutting process. As new aerospace equipment is developing toward the direction of higher flight safety, higher carrying capacity and longer flight distance [1], it is essential to further improve the performance of TWCCTRR. Nevertheless, the cutting process can no longer satisfy the manufacturing requirements of TWCCTRR utilized in new aerospace equipment because it fails to refine grains and destroys metal streamlines [2,3]. In this situation, it is also necessary to develop a new advanced manufacturing method to improve the performance of TWCCTRR further.

**Figure 1:**
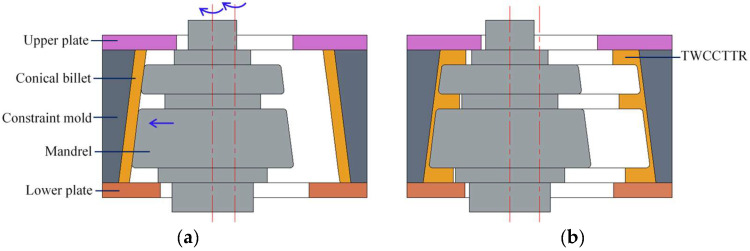
Principal diagram for the CRR of TWCCTRR [4]. (**a**) Start of CRR; (**b**) end of CRR.

Constraint ring rolling (CRR) is a new plastic forming technique, and it is very applicable for forming TWCCTRR [4]. In the CRR of TWCCTRR, the outer radius and axial height of conical billet do not change under the role of constraint mold and upper/lower plate, as shown in Figure 1, by which the forming stability can be guaranteed, and meanwhile, much material is enforced to flow axially. Through the coordinated motion of molds (i.e., both the constraint mold and upper/lower mold rotate along their axes, and the mandrel translates radially and meanwhile rotates along its axis), the conical billet is gradually formed into TWCCTRR, and consequently, TWCCTRR can obtain refined grains and complete material streamlines. Owning to the above principle, CRR has great potential in forming high-performance TWCCTRR.

Regarding the new CRR technology, the authors’ research team has conducted some basic investigations previously. Based on the FE simulation and experiment, the deformation mechanism and forming rules in the CRR of a thin-walled cylinder with two ring ribs were revealed [5]. Further, the prediction model of rib height in the CRR of a thin-walled cylinder with two ring ribs was constructed by mechanical modelling [6], and its reliability was verified by physical experiments. Using this model, the uncoordinated growth mechanisms of the two ribs were revealed. Compared to the CRR of a thin-walled cylinder with two ring ribs, the material flow is more complicated in the CRR of TWCCTRR, which may cause a shrinkage defect and unreasonable material flow (UMF) at the junction of the skin and the middle rib. Therefore, the effects of geometric parameters on the shrinkage defect in the CRR of TWCCTRR were analyzed, and the shrinkage defect was eliminated by optimizing geometric parameters [7]. In addition, the material flow prediction for the CRR of TWCCTRR was constructed and validated [4]. Using this material flow prediction model, the relationship between the conical angle of the skin, the position of the middle rib and the UMF in the CRR of TWCCTRR was established, and the material flow mechanism on the condition that UMF does not occur was also revealed [4]. It should be emphasized that UMF seriously weakens the mechanical properties of forgings [8], so UMF should be avoided in the CRR of TWCCTRR. However, UMF is prone to occur in the CRR of TWCCTRR due to uncoordinated material flow, and there is still no effective method of avoiding UMF.

In practice, material flow control is very critical to the forming quality of metallic components [9], and it is still widely studied. Until now, many investigations on material flow control in forming metallic components have been conducted. By adjusting extrusion parameters to narrow material flow deviation in the cross-section of the extruded profile with complicated geometry, the performance homogeneity of the extruded profile was improved [10]. It has been proven that both material anisotropy and loading mode are the main causes of anisotropy material flow in the forming of metallic plate, and thus, a new constitutive model considering time-varying *r*-value was developed by Yang et al. to improve the simulation accuracy of anisotropy material flow in metallic plate forming processes [11]. In addition, uncoordinated material flow easily promotes the occurrence of folding defects in metal forming, and the formation causes of folding defects in the plastic forming of thin-walled tubes were revealed by Li et al. and Meng et al. [12,13]. As is well known, the folding defect induced by uncoordinated material flow would directly make the product scrapped. Therefore, the universal prediction model for various types of folding defects in local loading forming of large, complicated components was developed and validated by Gao et al. [14,15]. 

Furthermore, the control method of folding defect was proposed by Gao et al. [16,17], by which the size limit of the formed component without folding defect was effectively lifted. Aside from the folding defect, the defects of concave and rib underfilling also easily occur in metal forming due to uncoordinated material flow, which seriously lowers the forming accuracy of the product. For the concave defect, Xia et al. revealed the formation cause of this defect in the flow forming of cylinder part with ribs by analyzing the stress and velocity fields around the concave defect [18]. For rib underfilling, scholars mainly adopted the approach of optimizing process parameters or billet shape to reasonably adjust material allocation so as to eliminate the defect of rib underfilling [19,20,21]. With the principle of differential material flow velocity, Zhou et al. proposed a new differential-velocity extrusion technique for manufacturing curved bars, and the relations between process parameters, material flow and the shape of the curved bar in differential-velocity extrusion forming were analyzed by combining analytical solution, FE simulation and experiment [22,23,24].

Summarily, the material flow control methods proposed in the previous studies were used to eliminate the defects of concave, folding and underfilling in metal forming, but they cannot be used to eliminate the defect of UMF. Therefore, this work mainly concentrates on the UMF problem in the CRR of TWCCTR. Firstly, the formation rules and cause of UMF in single-pass CRR of TWCCTRR are analyzed based on physical experiments and FE simulation. Then, the double-pass CRR process for forming TWCCTRR without UMF is proposed, and its feasibility is validated by FE simulation. Finally, the deformation rules of TWCCTRR in single-pass CRR and double-pass CRR are contrastively analyzed by FE simulation.

## 2. Analysis of Material Flow in Single-Pass CRR of TWCCTRR

### 2.1. Material and Geometric Parameters

**Figure 2:**
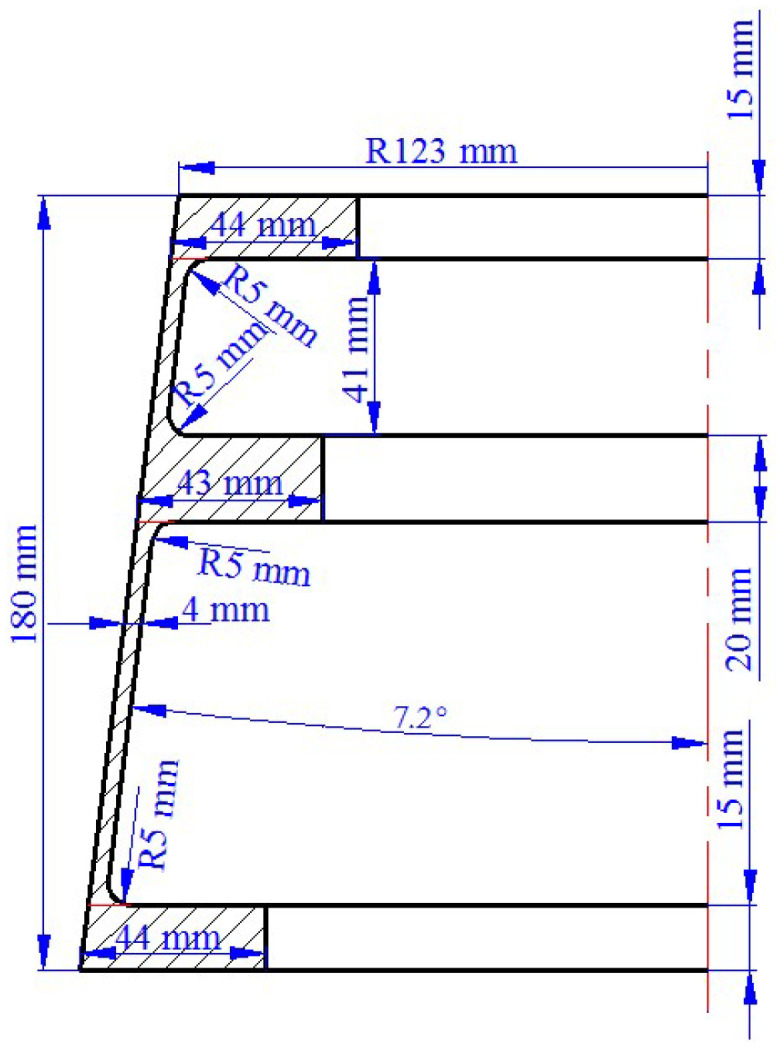
Dimensional diagram of typical TWCCTRR utilized in aerospace equipment.

The defect of UMF easily occurs in the single-pass CRR of TWCCTRR because of uncoordinated material flow in the axial direction. Therefore, a typical 7055 aluminum alloy TWCCTRR utilized in aerospace equipment is chosen as a research object to analyze material flow rules in single-pass CRR of TWCCTRR, the 1:1 dimension of which is shown in Figure 2. The outer diameter, axial height and conical angle of conical billet used for single-pass CRR of TWCCTRR are respectively the same as that of target TWCCTRR in Figure 2.

### 2.2. Physical Experiment

**Figure 3:**
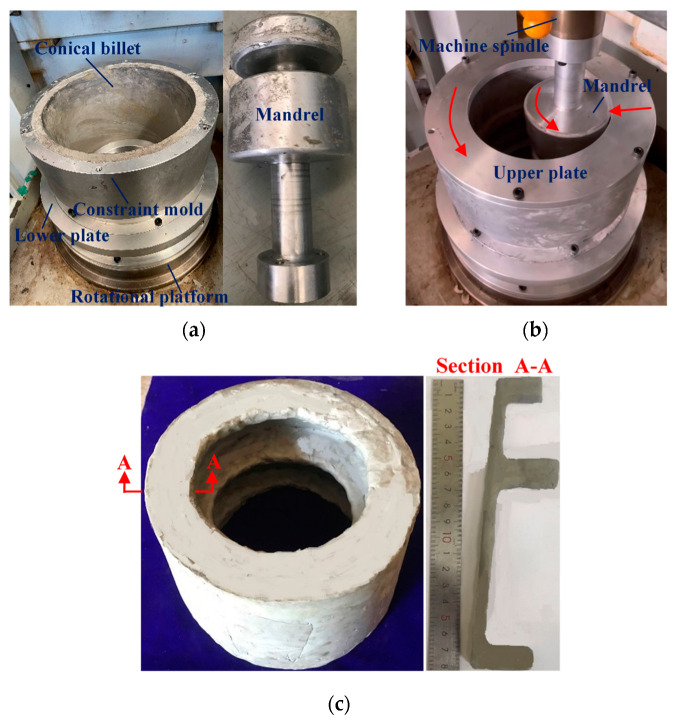
Physical experiment for single-pass CRR of TWCCTRR: (**a**) appearance of molds and billet; (**b**) experimental setup; (**c**) appearance of formed plasticine TWCCTRR.

Considering that the specific CRR equipment for forming large-scale aluminum alloy TWCCTRR is still lacking, a physical experiment of plasticine for single-pass CRR of TWCCTRR is conducted on a CNC gear shaper machine manufactured by Tianjin Machine tools Co., TLD of Tianjin, China. As shown in Figure 3a,b, a conical billet of plasticine with 13.6 mm in radial wall thickness is well matched with the conical cavity of constraint mold; that is, the outer circumferential surface of the conical billet contacts with the inner circumferential surface of constraint mold, and the axial height of conical billet is the same as that of constraint mold. In addition, the upper plate and lower plate are respectively installed on the upper and lower end surfaces of the constraint mold, and the lower plate is linked with the rotational platform of the machine. Moreover, the mandrel, linked with the spindle of the machine, makes contact with the inner surface of the conical billet, and the distance from the lower surface of the mandrel to the lower surface of the conical billet is 15 mm. To improve the material fluidity of the plasticine conical billet, the working surfaces of the molds are coated with resin lubricant in advance. For the physical experiment of the single-pass CRR, the feed speed and rotation speed of the constraint mold are 0.2 mm/s and 1.57 rad/s, respectively; the rotation speed of the mandrel is 3.14 rad/s and the key dimensions of molds are given in Table 1.

Figure 3c displays the appearance of formed TWCCTRR as the feed amount of constraint mold reaches 8 mm. It shows that the thickness of skin is greatly reduced, and the height of three ribs is greatly increased. Among the three ribs, the height of the rib at the middle part is the largest, and the height of the rib at the small part is the smallest. This phenomenon was also reflected in the single-pass CRR experiment of small-scale AA1050 TWCCTRR [4]. The above analysis illustrates that there exists an obvious gap in the amount of material flowing toward the rib at the large/middle/small part in single-pass CRR. 

### 2.3. Numerical Modeling and Analysis

To clearly analyze the material flow rules in single-pass CRR of TWCCTRR, a rigid visco-plastic FE model is established in DEFORM-3D software with the 11th version, and the reliability of FE model was validated in the literature [4]. In this FE model, all molds are defined as rigid bodies, and the conical billet is defined as a deformable body. To ensure the efficiency and accuracy of FE simulation, the conical billet is divided into 400,000 tetrahedral meshes with the mesh size of 0.5 mm~1.5 mm, and the auto-remeshing criteria with the relative interference depth of 0.7 is adopted to avoid mesh distortion in large deformation. The material of conical billet is annealed 7055 aluminum alloy fabricated by spray forming, and the constitutive model of this material considering material viscosity is obtained from the literature [25]. The temperature of the conical billet and molds is still 723 K, and the contact mode between the conical billet and molds is selected as constant shear friction. It was experimentally verified that the friction factor between the aluminum alloy billet and the steel molds in hot forming using the MoS_2_ lubricant is approximately 0.3 [26], so the friction factor in this FE model is set as 0.3. The rotation speed of the constraint mold is 21.8 rad/s. The mandrel passively rotates along its axis and, meanwhile, radially translates with a speed of 1 mm/s, and the total feed amount of the mandrel is 9.6 mm. 

**Figure 4:**
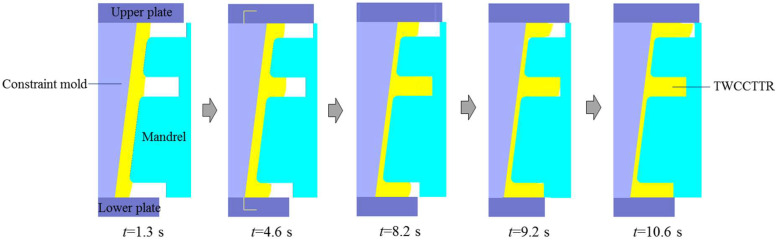
Shape revolution rules of deformed TWCCTRR during single-pass CRR of TWCCTRR.

Through FE simulation, the shape revolution rules of deformed TWCCTRR during single-pass CRR of TWCCTRR are displayed, as shown in Figure 4. As can be seen from Figure 4, with the radial feed of the mandrel, the rib at the middle part shows the fastest growth rate, while the rib at the small part shows the slowest growth rate. This is because the distance from the rib at the middle part to the rib at the large part is approximately twice that from the rib at the middle part to the rib at the small part. According to the literature [4], it is known that the amount of material flowing from the upper loading area of the skin toward the rib at the small part is much less than that of material flowing from the lower loading area of the skin to the rib at the large part, resulting in that the growth rate of the rib at the small part is smaller than that of the rib at the large part. On the other hand, the amount of material flowing from both the upper and lower loading areas of the skin to the rib at the middle part is slightly greater than that of material flowing from the lower loading area of the skin to the rib at the large part, and thus the growth rate of the rib at the middle part is slightly larger than that of the rib at the large part under the combined influences of ribs’ profile sizes. When the rolling time *t* = 8.2 s, the rib at the middle part is completely filled (i.e., the height of the rib at the middle part reaches its target value), while the other two ribs are not completely filled. Then, as the wall thickness of the skin further decreases, the height of the rib at the middle part remains unchanged, and the other two ribs continuously grow. When the rolling time *t* = 9.2 s, the rib at the large part is completely filled, while the rib at the small part is still not completely filled. Then, as the further feed of the mandrel, the height of the rib at the large/middle part remains unchanged, and thus, all the material within the upper and lower loading areas of the skin flows toward the rib at the small part, resulting in that the height of the rib at the small part quickly increases to its target value. It can also be observed from Figure 4 that during the single-pass CRR of TWCCTRR, macro defects such as wrinkling, shrinkage and underfilling do not occur. 

**Figure 5:**
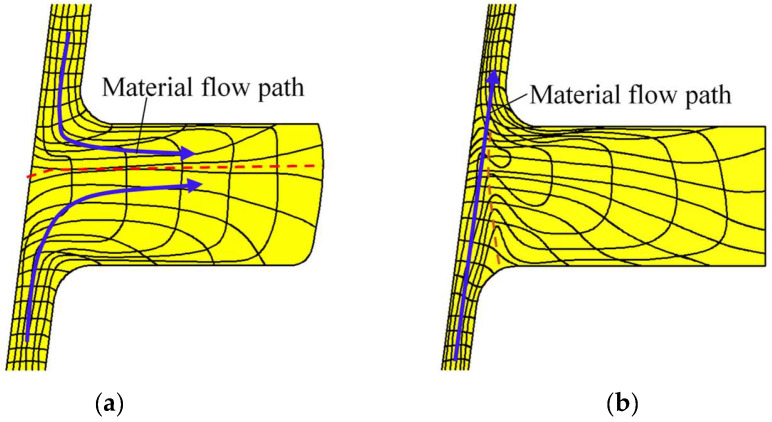
Distribution rules of material streamline around the rib at the middle part during single-pass CRR of TWCCTRR. (**a**) When the rolling time *t* = 8.2 s; (**b**) when the rolling time *t* = 10.6 s.

Notably, UMF does not occur around the two ribs located at the large and small parts in the CRR of TWCCTRR [2] because the material flows from the skin to these two ribs under the cooperative action of molds. However, UMF easily occurs around the rib located at the middle part because there exists a complicated competition relation between the material flow at the upper loading area of the skin and the material flow at the lower loading area of the skin. Therefore, it is necessary to analyze the evolution rules of material streamline around the rib at the middle part in the CRR of TWCCTRR. Based on the FE simulation, the distribution rules of material streamline around the rib at the middle part in single-pass CRR of TWCCTRR are revealed, as shown in Figure 5. 

When the rolling time *t* = 8.2 s, the rib at the middle part is not completely filled, and it can be observed from the shape of material streamlines (the black curved lines in this figure denote material streamlines) that the material at the upper and lower loading areas flows together to the rib at the middle part (the blue curved arrows in this figure denote material flow path), as shown in Figure 5a. Under the above material flow mode, the material from the upper and lower loading areas of the skin converges at this rib (the red dotted line denotes the convergence surface of the material), and thus, reasonable material streamlines are generated. Obviously, the convergence surface is closer to the upper surface of the rib at the middle part, which means that the amount of material flowing from the upper loading area to the rib at the middle part is far less than that of material flowing from the lower loading area to the rib at the middle part. This phenomenon can be explained by the fact that the upper loading area is much smaller than the lower loading area. When the rolling time *t* = 9.2 s, the two ribs at the middle and large parts are completely filled, while the rib at the small part is not completely filled, as shown in Figure 4. Therefore, the material at the upper and lower loading areas of the skin is forced to flow to the rib at the small part at this moment. It can be observed from Figure 5b that the material directly flows from the lower loading area of the skin to the upper loading area. Under this material flow mode, a long shear zone is generated at the junction of the skin and the rib located at the middle part (the orange dotted curved line in Figure 5b denotes the shear zone), in which material streamlines are disordered, resulting in the appearance of UMF. 

## 3. Process Design and Analysis for Double-Pass CRR of TWCCTRR

### 3.1. Design of Preformed Conical Billet

**Figure 6:**
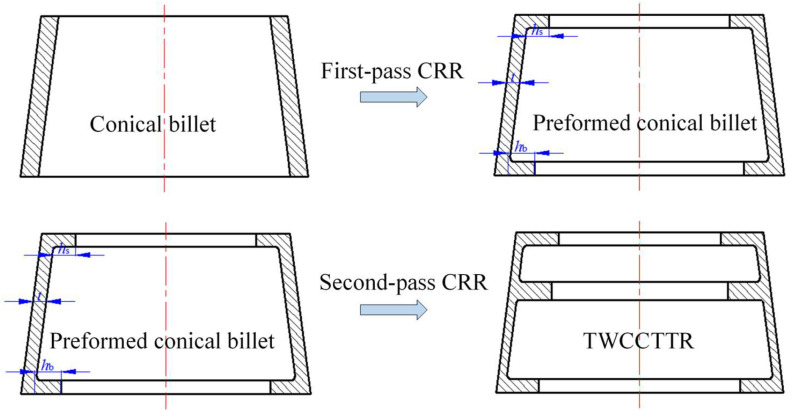
Process procedure for double-pass CRR of TWCCTRR.

From the above analysis, we found that the cause of UMF occurring in single-pass CRR of TWCCTRR is that the rib at the small part is completely filled, the latest among the three ribs. If the three ribs could be completely filled simultaneously, UMF may be avoided in the CRR of TWCCTRR. Motivated by this idea, the double-pass CRR process is proposed in this paper to form TWCCTRR without UMF; that is, a conical billet is rolled into a preformed conical billet that has two inner ring ribs (their widths are all 15 mm) at the small and large parts through first-pass CRR, and then the preformed conical billet is rolled into the target TWCCTRR through the second-pass CRR, as shown in Figure 6. Importantly, the height of the two ribs, as well as the wall thickness of the skin for the preformed conical billet, determine if the three ribs of TWCCTRR can be completely filled simultaneously in the second-pass CRR. Therefore, precisely designing the preformed conical billet is critical to avoiding UMF in the double-pass CRR of TWCCTRR. 

Based on the law of material volume constancy, it is deemed that the volume of the preformed conical billet is the same as that of target TWCCTRR shown in Figure 2. Accordingly, the quantitative relation between key dimensions of the preformed conical billet is determined, as expressed in Equation (1).

(1) $$\frac{10\left[{\left(124.9-t\right)}^{2}+\left(124.9-t\right)\left(143.8-t\right)+{\left(143.8-t\right)}^{2}\right]}{3}+{\left(124.9-t-h_{s}\right)}^{2}+{\left(145.7-t-h_{b}\right)}^{2}=17780$$

where *t* denotes the radial wall thickness of the preformed conical billet, *h*_s_ denotes the height of the rib at the small part of the preformed conical billet, and *h*_b_ denotes the height of the rib at the large part of the preformed conical billet. 

**Figure 7:**
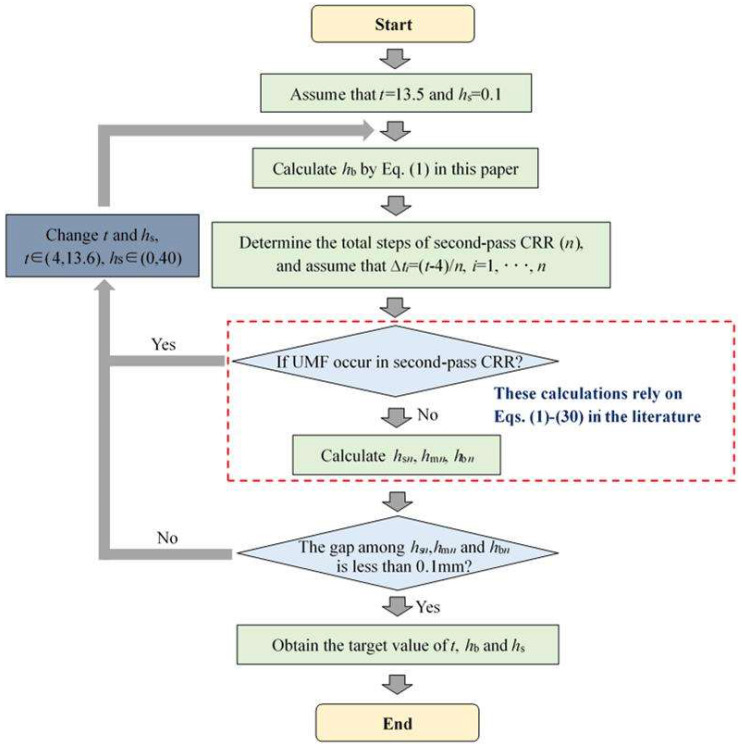
Calculation procedure of key parameters of preformed conical billet for the second-pass CRR [4].

To ensure that the three ribs of target TWCCTRR can be completely filled simultaneously, the precise calculation method of key parameters *t*, *h*_s_ and *h*_b_ is proposed, by which these key parameters can be quickly determined. As shown in Figure 7, the proposed calculation method mainly contains the following procedures: (1) assign an initial value for parameters *t* and *h*_s_, calculate *h*_b_ based on Equation (1), determine the total forming steps of the second-pass CRR (*n*), and assume that the reduction amount of radial wall thickness of the skin in the *i*th forming step (∆*t_i_*) is the same (*i* in Figure 7 denotes the number of forming step for the second-pass CRR); (2) in reference to Equations (1)–(30) in the literature [4], judge if UMF would occur in each forming step (once UMF occurs, immediately change the values of *t* and *h*_s_, and repeat the above procedures); (3) if UMF does not occur in each forming step, calculate the height of the three ribs in the last forming step by means of Equations (1)–(30) in the literature [4] (*h*_s*n*_, *h*_m*n*_ and *h*_b*n*_ respectively denote the height of the rib at the small, middle and large parts in the last forming step); (4) judge if the gap among *h*_s*n*_, *h*_m*n*_ and *h*_b*n*_ is less than 0.1 mm (once the gap is not less than 0.1 mm, immediately change the values of *t* and *h*_s_ and repeat the above procedures); (5) if the gap among *h*_s*n*_, *h*_m*n*_ and *h*_b*n*_ is less than 0.1 mm, output the final value of *t*, *h*_s_ and *h*_b_.

Based on the proposed calculation method of key parameters *t*, *h*_s_ and *h*_b_, it is determined that *t* = 11.2 mm, *h*_s_ = 22 mm and *h*_b_ = 11.2 mm. This means that the total reduction in the radial wall thickness of the skin in the first-pass CRR and the second-pass CRR is 2.4 mm and 7.2 mm, respectively. 

### 3.2. Analysis of Material Flow in Double-Pass CRR of TWCCTRR

To verify whether UMF in TWCCTRR can be effectively eliminated by the double-pass CRR process designed above, the FE simulation for double-pass CRR of TWCCTRR is also conducted based on DEFORM-3D software. In this FE simulation, the mandrel used for the first-pass CRR is different from that used for the second-pass CRR. There is no ring cavity at the middle part of the mandrel used for the first-pass CRR, and thus, the preformed conical billet with two ring ribs at the small and large parts can be formed as the first-pass CRR is accomplished. The shape of the mandrel for the second-pass CRR is the same as that of the mandrel for the single-pass CRR. The radial feed amount of the mandrel for the first-pass CRR and second-pass CRR is respectively 2.4 mm and 7.2 mm. Notably, the mandrel used for the second-pass CRR does not contact the skin of preformed conical billet during the first-pass CRR, and the mandrel used for the first-pass CRR does not contact the skin of deformed TWCCTRR during the second-pass CRR. When the first-pass CRR is ended, the mandrel used for the second-pass CRR begins to contact the skin of the preformed conical billet. The other boundary conditions in the FE simulation conducted for the double-pass CRR are all the same as those in the FE simulation conducted for the single-pass CRR.

**Figure 8:**
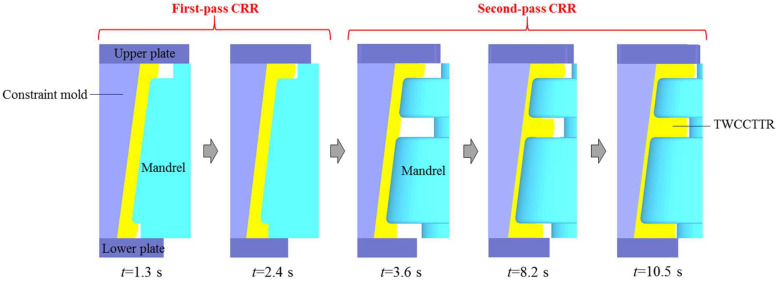
Shape revolution rules of TWCCTRR during the double-pass CRR of TWCCTRR.

Through FE simulation, the shape revolution rules of deformed TWCCTRR during double-pass CRR of TWCCTRR are displayed in Figure 8. In the stage of the first-pass CRR, the two ribs at the small and large parts almost grow simultaneously before they are completely filled, the cause of which has been explained in the literature [7]. When the rolling time *t* ≈ 1.5 s, the rib at the large part is completely filled firstly because the target height of this rib (11.2 mm) is much smaller than that of the rib at the small part (22 mm). Subsequently, as the wall thickness of the skin continues to decrease, the height of the rib at the large part remains unchanged because this rib is completely filled, and thus, the material in the loading area of the skin is forced to flow toward the rib at the small part. At the end of the first-pass CRR, the rib at the small part is basically completely filled (its height basically reaches 22 mm). Owning to the above material flow mode, UMF does not occur at the two ribs located at the large and small parts during the first-pass CRR [4].

In the stage of the second-pass CRR, the rib at the middle part grows fastest while the rib at the small part grows slowest. This can be explained as follows: since the height of the rib at the small part is much greater than that of the rib at the middle part, the resistance to upward flow of the material in the upper loading area of the skin (this area is located at the middle of these two ribs) is greater than the resistance to downward flow of the material in the upper loading area of the skin, resulting in that more material in the upper loading area of the skin flows toward the rib at the middle part. Similarly, since the height of the rib at the large part is much greater than that of the rib at the middle part, more material in the lower loading area of the skin (the two ends of this area are respectively linked with the ribs at the large and middle parts) flows toward the rib at the middle part, resulting in that the growth rate of the rib at the large rib is obviously smaller than that of the rib at the middle part. Since the upper loading area of the skin is much smaller than the lower loading area of the skin, the amount of material allocated from the upper loading area of the skin to the rib at the small part is much less than that of material allocated from the lower loading area of the skin to the rib at the large part, resulting in that the growth rate of the rib at the large part is obviously larger than that of the rib at the small part. For the above reasons, the height difference between the three ribs is gradually reduced with the radial feed of the mandrel, as shown in Figure 8, and the three ribs are nearly simultaneously completely filled at the end of the second-pass CRR. It can also be observed from Figure 8 that through the double-pass CRR process proposed in this paper, the surface quality of formed TWCCTRR is good (macro defects do not occur).

**Figure 9:**
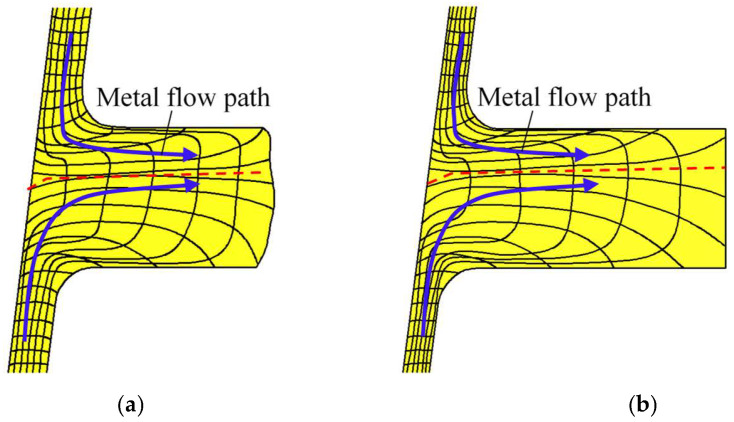
Distribution rules of material streamline around the rib at the middle part during double-pass CRR of TWCCTRR. (**a**) When the rolling time *t* = 8.2 s; (**b**) when the rolling time *t* = 10.6 s.

To verify whether UMF occurred during the second-pass CRR, the distribution rules of material streamlines when the rolling time *t* = 8.2 s and *t* = 10.6 s are displayed in Figure 9a,b, respectively. 

When the rolling time *t* = 8.2 s, the rib at the middle part is not completely filled, and it can be observed from Figure 9a (material streamlines are denoted by the black curved lines in this figure) that the material at the upper and lower loading areas flows together to the rib at the middle part. Before the rolling time *t* = 10.6 s, the rib at the middle part is still not completely filled, and it can be observed from Figure 9b that the material at the upper and lower loading areas still flows together to the rib at the middle part. Under the above material flow mode, the material from the upper and lower loading areas of the skin converges at the rib at the middle part (the convergence surface of the material is denoted by the red dotted line), and thus TWCCTRR without UMF is formed by the double-pass CRR process.

## 4. Contrastive Analysis of Deformation Behaviors in Single-Pass CRR and Double-Pass CRR

**Figure 10:**
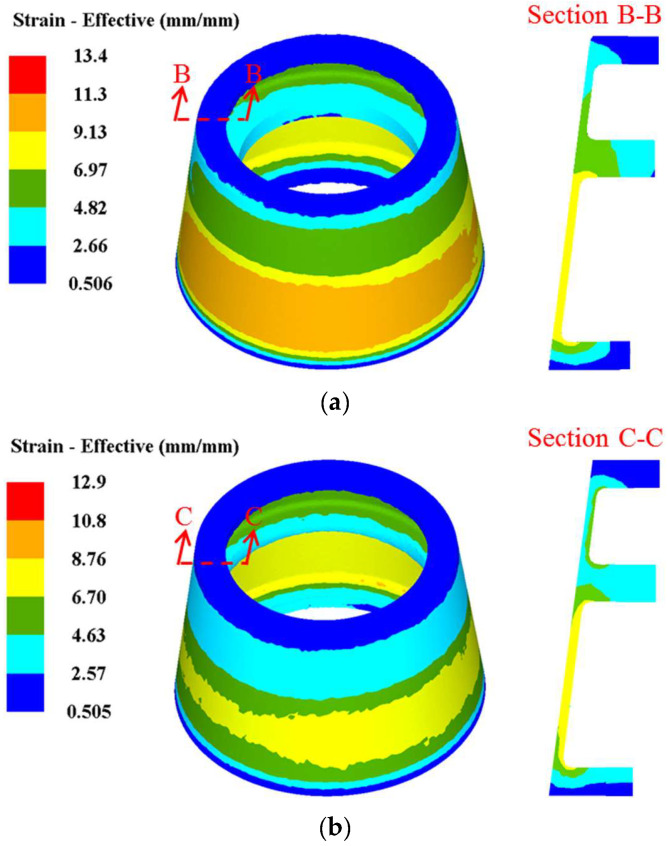
Distribution rules of effective strain in TWCCTRR. (**a**) when single-pass CRR is accomplished; (**b**) when double-pass CRR is accomplished.

Based on the FE simulation, the distribution rules of effective strain in TWCCTRR formed by single-pass/double-pass CRR are respectively displayed in Figure 10a,b. Figure 10a,b show that when single-pass CRR and double-pass CRR are accomplished, the distribution of effective strain along the axial/radial direction is highly uneven, and the distribution of effective strain along the circumferential direction is relatively uniform. The effective strain in the lower loading area of the skin is the maximum, and the effective strain around the end surfaces of the two ribs at the large and small parts is the minimum. From the bottom of the three ribs to their roofs, the effective strain basically presents a gradual decrement trend. Additionally, the effective strain in the upper loading area of the skin is significantly less than that in the lower loading area of the skin, and the effective strain around the roof of the rib at the middle part is much larger than that around the roofs of the two ribs at the large and small parts.

**Figure 11:**
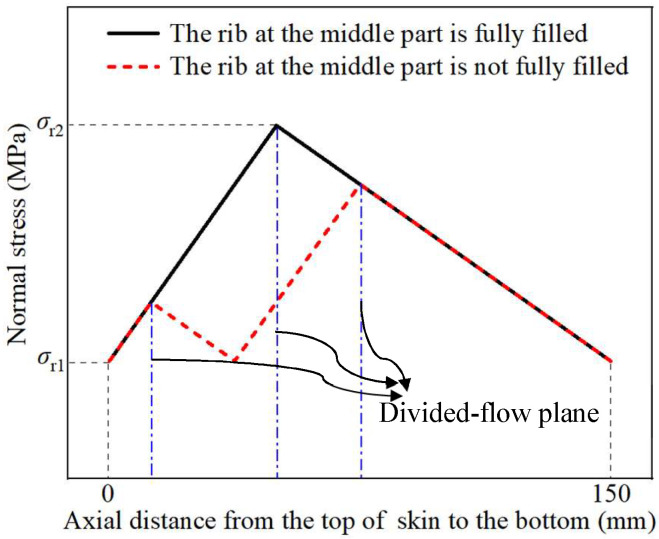
Schematic diagram of normal stress distribution in the loading area of the skin in the CRR of TWCCTRR.

Through comparing Figure 10a,b, it is found that the maximum effective strain in TWCCTRR formed by single-pass CRR is obviously larger than that in TWCCTRR formed by double-pass CRR, while the minimum effective strain in TWCCTRR formed by single-pass/double-pass CRR is basically the same. This demonstrates that in contrast to single-pass CRR, the plastic deformation in TWCCTRR formed by double-pass CRR is more uniform, which can be explained by the stress state of the loading area of the skin. In reference to the slab stress method [7], the schematic diagram of normal stress distribution in the loading area of the skin during the first-pass/second-pass CRR of TWCCTRR is drawn in Figure 11. It shows that when the rib at the middle part is not completely filled, the material in the upper and lower loading areas of the skin flows together to this rib, and thus, there exists a divided-flow plane in the upper/lower loading area of the skin (the normal stress on the divided-flow plane is the largest around the divided-flow plane). After the rib at the middle part is completely filled, a divided-flow plane only exists in the loading area of the skin. Figure 11 shows that with increasing the number of divided-flow planes in the skin, the maximum normal stress obviously decreases while the minimum normal stress remains unchanged. For the single-pass CRR of TWCCTRR, the number of divided-flow planes is changed from 1 to 2 after the rib at the large part is completely filled first. For the double-pass CRR of TWCCTRR, two divided-flow planes still exist in the skin because the three ribs are filled simultaneously. Based on the above analysis, it is illustrated that the distribution of normal stress on the skin in the final stage of the single-pass CRR is more uneven in contrast to double-pass CRR. Therefore, the distribution of plastic deformation in TWCCTRR formed by double-pass CRR is more uniform than that of first-pass CRR. 

**Figure 12:**
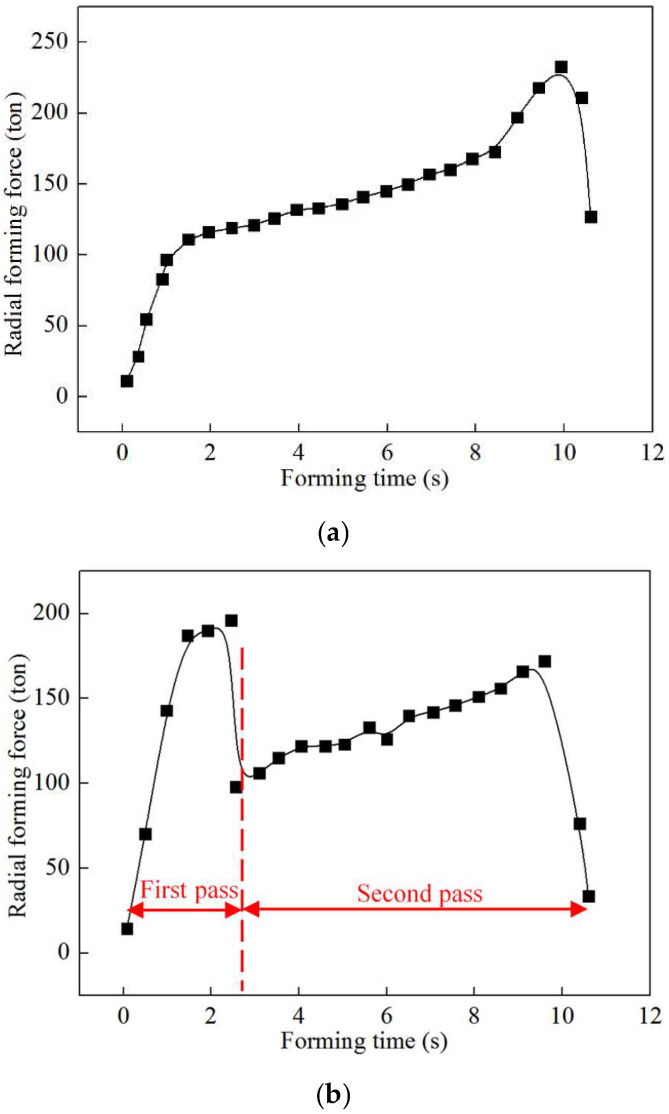
Evolution rules of radial forming force. (**a**) In single-pass CRR of TWCCTRR; (**b**) in double-pass CRR of TWCCTRR.

In addition, the evolution rules of radial forming force during the first-pass/double-pass CRR of TWCCTRR are respectively displayed in Figure 12a,b. As shown in Figure 12a, during the single-pass CRR, the radial forming force of the mandrel firstly quickly increases and then sharply decreases. This is because in the stage of primary forming (the mandrel continuously feeds in this stage), as the mandrel continuously feeds, the contact area between the mandrel and the skin gradually increases. Meanwhile, both the deformation-hardening effect and rib growth quickly increase material flow resistance, resulting in the rapid increment of radial forming force. However, in the final finishing stage (the mandrel does not feed in this stage), the contact area between the mandrel and the skin rapidly decreases, resulting in the sharp decrement of radial forming force. Notably, there is a sharp increase in the radial forming force after the feed amount of the mandrel reaches approximately 8.2 mm. This is because after the feed amount of the mandrel reaches approximately 8.2 mm, the rib at the middle part is completely filled, and meanwhile, the number of divided-flow planes changes from 2 to 1, resulting in the average normal stress in the loading area of the skin sharply increases as displayed in Figure 11. For the above cause, the radial forming force of the mandrel presents a sharp increment trend after the feed amount of the mandrel reaches approximately 8.2 mm.

As shown in Figure 12a, during double-pass CRR of TWCCTRR, the radial forming force of the mandrel shows a significant increase in the stage of the first-pass CRR, while the radial forming force firstly quickly increases and then quickly decreases in the stage of the second-pass CRR, which is mainly induced by the variation of the contact area between the mandrel and skin. Notably, in the transition stage between the first-pass CRR and second-pass CRR, there is a sharp decrement in the radial forming force. This is because the number of divided-flow planes changes from 1 to 2 in this stage, resulting in a sharp decrease in average normal stress in the loading area of the skin, as displayed in Figure 11. For the above cause, the radial forming force of the mandrel presents a sharp decrement trend in the transition stage between the first-pass CRR and second-pass CRR.

Figure 12a,b also show that the maximum radial forming force in single-pass CRR is obviously larger than that in double-pass CRR because the rib at the middle part is first completely filled in single-pass CRR, while the three ribs are basically filled at the same time in double-pass CRR.

## 5. Conclusions

Constraint ring rolling (CRR) is a new plastic forming technique for manufacturing thin-walled conical cylinders with three ring ribs (TWCCTRR). This paper aimed to analyze and solve the problem of unreasonable material flow (UMF) in the CRR of TWCCTRR by combining FE simulations and physical experiments with plasticine. The following main conclusions can be drawn.

(1) During the single-pass CRR of TWCCTRR, the growth of the rib at the middle part is the fastest, while the growth of the rib at the small part is the slowest among the three ribs at the large, middle and small parts. Once the two ribs at the large and middle parts are completely filled, the material in the upper and lower loading areas of the skin flows together to the rib at the small part, resulting in the occurrence of UMF around the rib at the middle part.
(2) The double-pass CRR process is proposed for forming TWCCTRR without UMF; that is, a conical billet is formed into the conical billet with two ring ribs by the first-pass CRR, and then the preformed conical billet with two ring ribs is formed into TWCCTRR by the second-pass CRR. The dimensions of the preformed conical billet with two ribs are critical to avoiding UMF in the second-pass CRR. Thus, a high-efficiency design method of preformed conical billet with two ribs is proposed, which ensures that the three ribs could be simultaneously and completely filled in the second-pass CRR to avoid UMF.
(3) In contrast to single-pass CRR, the deformation homogeneity of TWCCTRR is obviously improved, and meanwhile, the maximum radial forming force is reduced by approximately 15% in the double-pass CRR due to the variation in material flow mode.

In contrast to the single-pass CRR process, the double-pass CRR process increases fabrication costs due to the increment in processing time and number of molds. More importantly, the double-pass CRR process could obviously improve the mechanical properties of TWCCTRR in contrast to the single-pass CRR process due to the elimination of UMF. In the aerospace industry, the improvement in the mechanical properties of TWCCTRR is more important than the reduction in the fabrication cost of TWCCTRR. Thus, the proposed double-pass process CRR has good application prospects in fabricating high-performance thin-walled cylindrical components with the high ring ribs used in aerospace equipment. 

The material flow control methods proposed in the previous studies were used to eliminate the defects of concave, folding and underfilling in metal forming, but they cannot be used to eliminate the defect of UMF. The double-pass CRR process proposed in this paper can be used to eliminate the defect of UMF in the CRR of different types of cylindrical components with three or more ring ribs. This study could provide references for exploring the control methods of UMF in the forming of thin/thick plates with ribs.

In future, we plan to first develop a specific CRR equipment that can realize the forming of many types of thin-walled cylindrical components with high ribs used in aerospace equipment, then deeply investigate the regulation mechanisms of microstructure and mechanical properties in CRR of many types of thin-walled cylindrical components with high ribs, and finally put the CRR technology into engineering application. 

## Figures and Tables

**Table 1:** Key dimensions of molds in the physical experiment of the single-pass CRR of TWCCTRR.

Parameter	Value
Diameter of conical loading surface of mandrel at the large part	180 mm
Axial height of the conical loading surface of mandrel	150 mm
Width of cavity on the conical loading surface of mandrel	20 mm
Distance from the cavity on the conical loading surface to the upper surface of the conical loading surface of mandrel	41 mm
Conical angle of the conical loading surface of mandrel	7.2°
Inner diameter of lower plate	190 mm
Inner diameter of upper plate	146 mm

## Data Availability

The original contributions presented in this study are included in the article. Further inquiries can be directed to the corresponding authors.

## References

[1] Gao P., Gong Y., Ren Z., Zhan M. (2024). A new spinning-extrusion forming technology for the inner-ribbed component. Int. J. Mech. Sci..

[2] Park J., Hwang H. (2007). Preform design for precision forging of an asymmetric rib-web type component. J. Mater. Prcoessing Technol..

[3] Shan D., Xu W., Si C., Lu Y. (2007). Research on local loading method for an aluminum-alloy hatch with cross ribs and thin webs. J. Mater. Prcoessing Technol..

[4] Hua L., Tian D., Han X., Zhuang W. (2023). Modelling and analysis of material flowing behaviors in constraint ring rolling of tapered ring with thin wall and three high ribs. Chin. J. Aeronaut..

[5] Tian D., Han X., Hua L., Hu X. (2022). An innovative constraining ring rolling process for manufacturing conical rings with thin sterna and high ribs. Chin. J. Aeronaut..

[6] Feng W., Zhao P. (2024). Buckling defect optimization of constrained ring rolling of thin-walled conical rings with inner high ribs combining response surface method with FEM. Metals.

[7] Tian D., Han X., Hua L., Wang X., Chen F. (2022). Constraining ring rolling of thin-walled conical rings with transverse ribs. Int. J. Mech. Sci..

[8] Zheng Y., Liu D., Zhang Z., Yang Y., Ren L. (2017). The flow line evolution of hot open ACDR process for titanium alloy discs. Arch. Civ. Mech. Eng..

[9] Zhang Z., Chen Z., Xue Y., Zhang X., Wang Q. (2024). Investigation on low hydrostatic stress extrusion technology for forming of large thin-walled components with high ribs. Int. J. Mach. Tools Manuf..

[10] Zhang D., Xu H., Xu S., Tong F., Chen K., Li Z., Zuo J., Shu X. (2024). Material flow behavior and energy consumption model during the extrusion process of a 6063 aluminum alloy profile with complex cross-section. J. Mater. Res. Technol..

[11] Yang H., Zhang W., Zhuang X., Zhao Z. (2025). Experimental and modeling investigation on anisotropic plastic flow of metal plates under nonproportional loading conditions. Int. J. Solids Struct..

[12] Lin P., Guan Y., Kong P., Jiang S., Sun D., Guo S., Yan B., Feng H., Yang L., Zhang Y. (2024). Transfer mechanisms of folding defect for thin-walled tube end flanges formed by flaring-upsetting hybrid process. J. Manuf. Process..

[13] Meng Y., Yu Z., Zhao Y. (2024). Folding defects mechanism of aluminum alloy thin-walled stiffened cylinders during flow forming. Thin-Walled Struct..

[14] Gao P., Yang H., Fan X., Lei P., Meng M. (2014). Prediction of folding defect in transitional region during local loading forming of Titanium alloy large-scale rib-web component. Procedia Eng..

[15] Gao P., Fei M., Fan X., Wang S., Li Y., Xing L., Wei K., Zhan M., Zhou T., Keyim Z. (2019). Prediction of the folding defect in die forging: A versatile approach for three typical types of folding defects. J. Manuf. Process..

[16] Gao P., Yang H., Fan X., Lei P. (2015). Forming defects control in transitional region during isothermal local loading of Ti-alloy rib-web component. Int. J. Adv. Manuf. Technol..

[17] Gao P., Li X., Yang H., Fan X., Lei Z. (2017). Improving the process forming limit considering forming defects in the transitional region in local loading forming of Ti-alloy rib-web components. Chin. J. Aeronaut..

[18] Xia Q., Long J., Xiao G., Yuan S., Qin Y. (2021). Deformation mechanism of ZK61 magnesium alloy cylindrical parts with longitudinal inner ribs during hot backward flow forming. J. Mater. Process. Technol..

[19] Zhu X., Liu D., Yang Y., Hu Y., Liu G., Wang Y. (2016). Effects of blank dimension on forming characteristics during conical-section ring rolling of Inco718 alloy. Int. J. Adv. Manuf. Technol..

[20] Deng J., Mao H. (2015). A blank optimization design method for three-roll cross rolling of complex-groove and small-hole ring. Int. J. Mech. Sci..

[21] Zhuang W., Hua L., Han X., Zheng F. (2017). Design and hot forging manufacturing of non-circular spur bevel gear. Int. J. Mech. Sci..

[22] Zhou W., Lin J., Dean T., Wang L. (2018). Analysis and modelling of a novel process for extruding curved metal alloy profiles. Int. J. Mech. Sci..

[23] Zhou W., Lin J., Dean T., Wang L. (2018). Feasibility stumolds of a novel extrusion process for curved profiles: Experimentation and modelling. Int. J. Mach. Tools Manuf..

[24] Zhou W., Yu J., Lin J., Dean T. (2019). Manufacturing a curved profile with fine grains and high strength by differential velocity sideways extrusion. Int. J. Mach. Tools Manuf..

[25] Tian D., Han X., Hua L., Huang B., Yang S. (2020). A novel process for axial closed extrusion of ring part with mesh-like ribs. Int. J. Mech. Sci..

[26] Zong Y., Chen L., Zhao Z., Shan D. (2014). Flow lines, microstructure, and mechanical properties of flow control formed 4032 aluminum alloy. Mater. Manuf. Process..

